# CAR T cell-induced interferon gamma enhances MHC class I expression and sensitizes neuroblastoma to TCR-engineered T cell therapy

**DOI:** 10.3389/fimmu.2026.1901504

**Published:** 2026-09-18

**Authors:** Alice Vitali, Jennie Padlo, Carlotta Caramel, Lena Andersch, Laura M. Hartmann, Jonas Kath, Laura Grunewald, Silke Schwiebert, Anika Winkler, Efrosiinia Talalai, Tabea Biereder, Judith Wienke, Jan J. Molenaar, Michael Launspach, Catharina Delebinski, Anton Kern, Kathy Astrahantseff, Dimitrios L. Wagner, Gerald Willimsky, Angelika Eggert, Thomas Blankenstein, Kathleen Anders, Annette Künkele

**Affiliations:** 1 Charité – University Medical Center Berlin, corporate member of Freie Universität Berlin and Humboldt-Universität zu Berlin, Department of Paediatric Oncology and Haematology, Berlin, Germany; 2 German Cancer Consortium (DKTK), Partner site Berlin, and German Cancer Research Center (DKFZ), Heidelberg, Germany; 3 Berlin Institute of Health at Charité – Universitätsmedizin Berlin, BIH Center for Regenerative Therapies, Berlin, Germany; 4 Princess Máxima Center for Pediatric Oncology, Utrecht, Netherlands; 5 Department of Pharmaceutical Sciences, Utrecht University, Utrecht, Netherlands; 6 Institute of Tumor Immunology, Charité - Universitätsmedizin Berlin, Berlin, Germany; 7 Charité – University Medical Center Berlin, corporate member of Freie Universität Berlin and Humboldt-Universität zu Berlin, Institute of Tumor Immunology, Berlin, Germany; 8 University Hospital Essen, Essen, Germany

**Keywords:** antigen loss, MHC class I low, neuroblastoma, T cell therapy, TCR T cell resistance, tumor heterogeneity

## Abstract

**Background:**

T cell receptor (TCR)-engineered T cells target antigens presented on major histocompatibility complex (MHC) class I molecules. MHC class I expression is often low in childhood tumors such as neuroblastoma but can be stimulated by interferon gamma (IFNγ). Chimeric antigen receptor (CAR) T cell effector function, including IFNγ production, is MHC class I-independent.

**Aim:**

We aimed to enhance neuroblastoma immunotherapy by combining CAR- and TCR-engineered T cells. Our strategy exploits CAR T cell-derived effector molecules, including IFNγ, to increase tumor cell MHC class I expression and thereby enhance subsequent TCR T cell activity.

**Methods:**

IFNγ-mediated induction and persistence of MHC class I expression were analyzed in neuroblastoma cell lines by flow cytometry. Primary human T cells were engineered to express either a high-affinity TCR targeting the cancer testis antigen NY-ESO-1 or an L1CAM-targeted CAR. Cytotoxic activity of CAR and TCR T cells, applied alone or sequentially, was quantified against LAN-1 neuroblastoma cells using IncuCyte-based real-time killing assays. To model heterogeneous antigen expression, mixed tumor populations containing L1CAM-positive and CRISPR/Cas9-generated L1CAM-knockout cells were analyzed. *In vivo* MHC class I upregulation was assessed in a human tumor xenograft model following treatment with human L1CAM-CAR T cells and in a syngeneic tumor model following treatment with GD2-CAR T cells.

**Results:**

NY-ESO-1 TCR T cells alone showed limited cytotoxicity against LAN-1 cells *in vitro*, whereas L1CAM-CAR T cell pretreatment increased MHC class I expression and enhanced subsequent TCR-mediated killing. In tumor cells with heterogeneous CAR antigen expression, CAR T cell recognition of antigen-positive cells induced HLA-A*02 upregulation across the tumor population, enabling subsequent NY-ESO-1 TCR T cell recognition of cells not directly recognized by CAR T cells. Exploratory *in vivo* analyses further showed increased tumor MHC class I expression following CAR T cell treatment, providing preliminary evidence of CAR T cell-mediated inflammatory priming *in vivo*.

**Conclusion:**

Sequential CAR- and TCR-engineered T cell therapy may help overcome limitations imposed by low MHC class I and heterogeneous antigen expression in neuroblastoma, supporting further investigation of this combinatorial strategy.

## Introduction

Neuroblastoma is the most common extracranial solid tumor in children and remains a major therapeutic challenge in high-risk disease (1). Accordingly, there is a strong need for alternative therapeutic strategies to improve outcomes in children with chemotherapy-refractory or high-risk neuroblastoma. TCR-engineered T cell therapy represents a promising option, as expression of well characterized tumor antigens, including NY-ESO-1, MAGE-A1, and PRAME has been demonstrated in neuroblastoma (2–8).

Neuroblastoma originates from neural crest-derived sympathetic precursor cells arrested in early neuronal differentiation, a developmental state characterized by intrinsically reduced MHC class I expression (9, 10). This phenotype, which is particularly pronounced in high-risk disease (11), poses a major challenge for effective TCR-based immunotherapy due to the insufficient presentation of target MHC:peptide complexes on the tumor cell surface. Neuroblastomas retain the ability to upregulate MHC class I in response to IFNγ (3). This suggests that the TCR-targetable phenotype of neuroblastoma may be inducible under inflammatory conditions.

Coexisting tumor cell populations with diverse antigen expression profiles creates heterogeneity that forms a second critical obstacle to effective adoptive cell therapy. This heterogeneity is especially pronounced due to the developmental plasticity of neuroblastoma (12), which results in variable expression of therapeutic target antigens, exemplified by the transition of GD2-positive adrenergic tumor cells into GD2-negative mesenchymal-like states following GD2-targeting antibody therapy (13).

To address the intrinsic MHC class I deficiency in neuroblastoma, and challenges posed by tumor heterogeneity, we propose a sequential immunotherapy strategy that leverages established IFNγ-mediated upregulation of MHC class I. MHC-independent tumor-targeting CAR T cells are applied first to induce local IFNγ production, thereby restoring or enhancing MHC class I expression on tumor cells and enabling subsequent effective recognition by TCR-engineered T cells. Implementation of a dual-product approach poses practical challenges, particularly in heavily pretreated pediatric patients, from whom sufficient numbers of functional autologous T cells are often difficult to obtain. The use of T cells derived from healthy donors may therefore represent a feasible alternative, enabling the generation of a second T cell product with improved cellular fitness and more consistent product quality and scalability. However, the use of allogeneic T cells typically requires extensive genetic modification to prevent graft-versus-host disease and to avoid rapid elimination by the host immune system. This introduces additional risks related to complex genome engineering, including potential off-target effects, chromosomal rearrangements, and impaired cell fitness. A less extensively engineered allogeneic product - limited to CAR knock-in into the T cell receptor alpha constant (*TRAC*) locus without additional modifications such as MHC class I knockout - may mitigate these engineering-associated risks, but is expected to exhibit reduced persistence due to host immune rejection *in vivo*. Therefore, it remains unknown whether such a minimally edited CAR T cell product can provide sufficient inflammatory signaling, in particular IFNγ release, to induce MHC class I upregulation on neuroblastoma cells *in vivo* and thereby effectively enable subsequent TCR-mediated tumor recognition.

We evaluated a CAR/TCR combinatorial strategy *in vitro* using the LAN-1 neuroblastoma cell line as a representative model. L1CAM-targeting CAR T cells (14, 15) were combined with NY-ESO-1-specific TCR-engineered T cells (16, 17) that target an HLA-A*02:01-restricted NY-ESO-1_157-165_ (SLLMWITQC) epitope. Pretreatment of LAN-1 cultures with L1CAM-CAR T cells resulted in upregulation of MHC class I expression, even when CAR target antigen expression was restricted to a subset of tumor cells. While NY-ESO-1-specific TCR T cells alone were unable to efficiently eliminate LAN-1 cells, combined treatment with L1CAM-CAR T cells restored their cytotoxic activity *in vitro*. The ability of L1CAM-targeting CAR T cells to induce MHC class I upregulation on tumor cells was further confirmed *in vivo* in mice bearing LAN-1 xenografts. To further address the translational feasibility of a two-product strategy, we performed an exploratory assessment of whether minimally edited allogeneic GD2-CAR T cells could provide sufficient inflammatory signaling within tumors of immunocompetent mice. This study introduces a proof-of-concept combinatorial CAR/TCR strategy designed to address two major barriers in neuroblastoma - its intrinsic MHC class I deficiency and antigen heterogeneity - thereby enabling more effective T cell-based immunotherapy for this currently difficult-to-treat pediatric malignancy.

## Materials and methods

### Animals

NOD.Cg-Prkdc^scid Il2rg^tm1Wjl/SzJ (NSG) mice were obtained from The Jackson Laboratory (Bar Harbor, ME, USA) and were kindly provided by G. Willimsky (MDC, Berlin) for use in this study. A/J mice, and OT-1 TCR transgenic Rag2^-/-^ mice were bred in-house. Upon arrival at the experimental facility, mice were allowed a 7-day acclimation period. Mice were housed in groups under controlled conditions: temperature 22 ± 2 °C, humidity 55 ± 10%, and a 12 h light/dark cycle.

### Cell lines and culture conditions

All cell lines were maintained at 37 °C in a humidified incubator with 5% CO_2_. The SH-EP, SK-N-AS, SK-N-DZ, SK-N-SH, CHLA-20, SJNB-8, GI-ME-N, and NXS2 (murine) neuroblastoma cell lines and the LentiX 293T lentiviral vector packaging cell line were cultured in Dulbecco’s Modified Eagle’s Medium (DMEM; Thermo Fisher Scientific, Waltham, MA, USA) supplemented with 10% heat-inactivated fetal calf serum (Sigma-Aldrich, St. Louis, MO, USA). The H9 CD4+ T cell line and the NB-1691, SK-N-FI, IMR-32, KELLY, NB69, NMB, SH-SY-5Y, NGP, SK-N-BE(2), NB-1643, CHP-212, CHP-901, NB-1, NLF, LAN-6, SMS-KAN, NBL-S and LAN-1 neuroblastoma cell lines were cultured in Roswell Park Memorial Institute Medium (RPMI-1640, Thermo Fisher Scientific, Waltham, MA, USA) supplemented with 10% heat-inactivated fetal calf serum. The CHLA-90 and NB-Ebc-1 neuroblastoma cell lines were cultured with Isocove’s Modified Dulbecco’s Medium (IMDM, Thermo Fisher Scientific, Waltham, MA, USA) supplemented with 20% heat-inactivated fetal calf serum. All human cell lines were authenticated by STR analysis using the PowerPlex^®^ 16 System (Promega, Madison, WI, USA) and fragment length analysis of PCR products was performed by Eurofins Genomics (Ebersberg, Germany). Mycoplasma contamination was regularly tested using cell-based colorimetric HEK-Blue detection (InvivoGen, San Diego, CA, USA). Additional information on the tumor cell lines used in this study, including their respective applications, is provided in the **Supplementary Material** (**Supplementary Table 1**).

### *L1CAM* knockout by CRISPR/Cas9

To generate an L1CAM-deficient LAN-1 cell line, cells were transfected with a plasmid encoding Cas9, an *L1CAM*-targeting single-guide RNA (GCCTCGGGGATCTGGATAAGC), and a puromycin resistance cassette. LAN-1 cells were transfected using Effectene Transfection Reagent (Cat. No. 301425, QIAGEN, Hilden, Deutschland) following the manufacturer’s instructions. For each transfection, 1 µg plasmid DNA was used. One day after transfection, the medium was replaced, and on day 2 successfully transfected cells were selected with 0.5 µg/mL puromycin (Merck, Darmstadt, Germany) for 3 days. As a negative selection control, cells were transfected with a plasmid lacking the puromycin resistance gene. On day 10, knockout (KO) efficiency was analyzed by flow cytometry. Single-cell clones were generated to establish the LAN-1 L1CAM KO cell line.

### Generation of human TCR and CAR constructs and lentiviral vector production

The L1CAM-CAR (14, 15) and NY-ESO-1 TCR (16) were cloned into the self-inactivating (SIN) epHIV7 lentiviral vector plasmid. The CAR construct includes a single-chain variable fragment (scFv, clone CE7 (18)) targeting L1CAM, an IgG4 hinge, a CD28 transmembrane and co-stimulatory endodomain, and a CD3ζ signaling domain. This construct is co-expressed with a truncated epidermal growth factor receptor (EGFRt), positioned downstream of the receptor sequence and linked via a self-cleaving 2A peptide (T2A). This design enables the coordinated expression of the CAR and EGFRt from a single transcript, allowing for efficient detection and immunomagnetic enrichment of transduced T cells using EGFRt as a selectable surface marker. The TCR construct was derived from a transgenic mouse model expressing the human TCR α and β loci in the context of human HLA-A*02:01 (16). The receptor recognizes the HLA-A*02:01-restricted NY-ESO-1_157–165_ epitope (SLLMWITQC), but has also been shown to recognize this epitope in the context of at least two additional HLA-A*02 subtypes (17). The construct includes murine T cell receptor constant alpha (TRAC) and beta (TRBC) chains, each carrying a cysteine mutation (TRAC: T47C; TRBC: S54C), resulting in an additional disulfide bridge, which enhances correct α/β chain pairing. Lentiviral vectors were produced by transfecting Lenti-X 293T packaging cells with the CAR or TCR plasmid, along with packaging plasmids encoding gag/pol and rev, together with an envelope plasmid encoding VSV-G using the CalPhos Mammalian Transfection Kit (Cat. No. #631312, Takara Bio Inc., Shiga, Japan). Viral supernatants were harvested 72 h post-transfection, filtered (0.45 µm), concentrated by ultracentrifugation, and stored at −80 °C. Functional lentiviral titers were determined by transducing H9 target cells with serial dilutions of viral supernatant. Transduction efficiency was assessed by flow cytometry, and functional titers were calculated as: Titer (TU/mL) = (F × N × D)/V, where F represents the fraction of transduced cells, N the number of target cells, D the dilution factor, and V the volume of viral inoculum.

### Generation of lentivirally engineered human TCR and CAR T cells

A schematic illustrating the workflow for generating human TCR/CAR-engineered T cells is provided in **Supplementary Figure 2D**. For NY-ESO-1 TCR generation, CD8^+^ T cells were used, whereas CD3^+^ T cells were used for L1CAM-CAR T cell generation. T cell subsets were isolated from human peripheral blood mononuclear cells (PBMCs) of healthy donors using magnetic-activated cell sorting (MACS; CD8^+^ MicroBeads or Pan T Cell Isolation Kit for CD3^+^ selection; Miltenyi Biotec, Bergisch Gladbach, Germany, Cat. No. 130-045–201 and 130-096-535). CD8+ and CD3+ T cells were activated with activation beads (Miltenyi Biotec, Cat. No. 130-091-441) at a 1:1 bead-to-cell ratio. On day 3 post-activation, cells were transduced with lentiviral supernatant at a multiplicity of infection (MOI) of 3-5. CD8^+^ NY-ESO-1 T cells and CD3+ L1CAM-CAR T cells were expanded with 0.5 ng/mL IL-15 (Miltenyi Biotec, Cat. No. 130-095-765) and either 50 U/mL IL-2 (Novartis, Basel, Switzerland) or 10 ng/mL IL-7 (Miltenyi Biotec, Cat. No. 130-095-363), respectively. On day 14–16 after transduction, the NY-ESO-1 TCR-expressing T cell subset was enriched by MACS using a PE-conjugated anti-mouse TCR β constant region antibody (BioLegend, San Diego, CA, USA, Cat. No. 109208) in combination with anti-PE microbeads (Miltenyi Biotec, Cat. No. 130-105-639). Successfully transduced CAR T cells were enriched by MACS using PE-conjugated cetuximab (anti-EGFR) and anti-PE microbeads. Donor-matched untransduced CD8^+^ and CD3^+^ T cells were used as negative controls for functional assays. Following sorting, CAR and untransduced control T cells were stimulated with freshly thawed, irradiated allogeneic PBMCs (35 Gy) and irradiated EBV-transformed lymphoblastoid feeder cells (TM-LCL; 80 Gy), and OKT3 (anti-CD3 antibody, 30 ng/mL; Miltenyi Biotec, Cat. No. 130-093-377) and expanded for 13–15 days using a rapid expansion protocol (14).

### Generation of targeted non-virally engineered CAR T cells

Primary human T cells, enriched by CD3+ MACS sort, were subjected to non-viral CRISPR/Cas9-mediated CAR knock-in into the *TRAC* locus based on previously published protocols (19, 20). Briefly, chemically modified *TRAC*-targeting sgRNA (GAGAAUCAAAAUCGGUGAAU) (21) was complexed with recombinant SpCas9 protein to form ribonucleoprotein complexes. Double-stranded homology-directed repair (HDR) donor templates encoding the L1CAM-CAR transgene flanked by *TRAC* homology arms were generated by PCR amplification from plasmid templates and purified using AMPure XP beads. Activated T cells were electroporated with Cas9 RNPs and dsDNA HDR templates in 20 µL electroporation reactions using the Lonza 4D-Nucleofector system (Lonza, Basel, Switzerland) and program EH-115. After electroporation, 100 µL pre-warmed T cell culture medium was added directly to each 20 µL reaction, followed by a 10 min incubation at 37 °C. Cells were then resuspended and transferred into three volumes of T cell culture medium for expansion. For the first 24 h after electroporation, this expansion medium was supplemented with 0.5 µM Alt-R HDR Enhancer V2 (Integrated DNA Technologies, Coralville, IA, USA). To prevent CAR-mediated activation and differentiation during *ex vivo* expansion, 100 nM dasatinib was added throughout expansion and removed 24 h before functional assays, as previously described (22, 23). CD4/CD8 ratio was assessed before functional evaluation (**Supplementary Figure 3B**).

### Generation of retrovirus for murine CAR T cell generation

Retroviral particles encoding the murine GD2 CAR construct were produced using Lenti-X 293T cells. Cells were seeded at a density of 15 × 10^6^ cells per T175 flask and transfected at 70-80% confluency using the Lipofectamine 3000 Transfection Kit (Thermo Fisher Scientific) according to the manufacturer’s instructions. After 16–20 h, the medium was replaced with fresh complete medium. Viral supernatants were harvested 48 h post-transfection, filtered through a 0.45 µm filter, and used immediately for transduction as described below.

### Transduction of murine splenocytes

Spleens from A/J and OT-1 TCR transgenic mice were harvested, mechanically dissociated through a 40 µm strainer, and rinsed with medium. Red blood cells were lysed using ACK lysis buffer, and cells were washed and passed again through a 40 µm strainer to obtain single-cell suspensions. Splenocytes were activated overnight with murine Dynabeads (Thermo Fisher Scientific, cat. no. 11453D) at a 2:1 bead-to-cell ratio. The following day, non-tissue culture-treated plates were coated with Retronectin (20 µg/mL, Takara Bio) for 2 h at room temperature, washed with phosphate-buffered saline, and blocked with 2% bovine serum albumin for 30 min. Plates were loaded with 1 mL viral supernatant and spinoculated (1.5 h at 32 °C, 3000 × g). After removal of supernatant, 1 mL T cell suspension (1 × 10^6^ cells/mL) and 0.5 mL viral supernatant were added for a second spinoculation (30 min at 800 × g). Cells were incubated overnight before addition of medium supplemented with human IL-2 (50 U/mL; Novartis) for OT-1-derived T cells or human IL-7 (5 ng/mL; cat. No. 130-095-362, Miltenyi Biotec) and human IL-15 (0.5 ng/mL; cat. No. 130-095-765, Miltenyi Biotec) for A/J-derived T cells (24). Cells were maintained at 0.5-0.8 × 10^6^ cells/mL with cytokine supplementation every 2–3 days. Transduction efficiency was assessed by flow cytometry on day 6, and cells were cryopreserved on day 10 in heat-inactivated fetal calf serum with 10% dimethyl sulfoxide.

### Flow cytometric marker and antigen detection

Cell surface expression of CD3 (BioLegend, Cat. No. 300324), CD8 (Cat. No. 301041), NY-ESO-1 TCR (detected via murine TRBC; Cat. No. 109208), HLA-A*02 (Cat. No. 343327), and HLA-A,B,C (Cat. No. 311430) was assessed using fluorophore-conjugated monoclonal antibodies from BioLegend. L1CAM was detected using an antibody from Miltenyi Biotec (Cat. No. 130-100-691), and EGFRt was detected using an antibody from R&D Systems (Minneapolis, MN, USA, Cat. No. FAB9577P). Activation markers were analyzed using BioLegend antibodies against CD69 (Cat. No. 310926), CD137 (4-1BB; Cat. No. 309822), and CD25 (Cat. No. 302611). Flow cytometry was performed on a Fortessa X-20 (BD Biosciences, Franklin Lakes, NJ, USA) and data processed using FlowJo software V10.10.0 (Tree Star Inc., Ashland, OR, USA). Dead cells were excluded from analyses using LIVE/DEAD™ Fixable Near IR (780) Viability Kit (Life Technologies, Carlsbad, CA, USA, Cat. No. L34992).

### Functional assays

To assess NY-ESO-1 TCR T cell functionality against LAN-1 *in vitro*, tumor cells were pre-incubated with 10 ng/mL IFNγ (PeproTech, Cranbury, New Jersey, USA) for 24 h. After washing, tumor cells were seeded and allowed to adhere for 3–5 h. NY-ESO-1 TCR T cells were then added at an effector-to-target (E:T) ratio of 1:1. As a positive control, IFNγ-treated neuroblastoma cells were pulsed with NY-ESO-1_157–165_ peptide (SLLMWITQC; Peptides & Elephants, Hennigsdorf, Brandenburg, Germany) for 30 min at 10 nM prior to seeding. For combined functionality assays, LAN-1 cells were seeded and allowed to adhere for 3–5 h before addition of L1CAM-CAR- T cells at an E:T ratio of 1:1. To enable subsequent identification of CAR T cells in the co-culture by flow cytometry, CAR T cells were labeled with CellTrace Violet (CTV; Thermo Fisher Scientific) prior to co-culture according to the manufacturer’s instructions. 24 h later, the same amount of NY-ESO-1 TCR-engineered T cells were added. T cell-mediated cytotoxicity was monitored using an IncuCyte^®^ S3 system (Sartorius, Göttingen, Germany). For visualization of tumor cell subsets, parental LAN-1 cells expressing a nuclear-localization signal (NLS)-containing mKate (NLS-mKate) and LAN-1 L1CAM KO cells expressing a NLS-GFP were used. Both tumor cell populations were mixed at a 1:1 ratio prior to addition of CAR- or TCR-engineered T cells. Data were analyzed using IncuCyte^®^ Software (Rev2, 2022; Sartorius, Germany). Surface expression of activation markers on NY-ESO-1 TCR-engineered T cells was assessed by flow cytometry 24 h after initiation of coculture. Supernatants were collected 24 h after coculture initiation and stored at −80 °C for cytokine analysis. IL-2 (BioLegend, Cat. No. 431816) and IFNγ (BioLegend, Cat. No. 430116) levels were quantified by ELISA according to the manufacturer’s instructions using an EPOCH microplate reader (BioTek Instruments, Winooski, VT, USA).

### Xenograft mouse model

Age- and sex-matched NSG mice were subcutaneously injected in the right flank with 1 × 10^7^ LAN-1 cells in 50 µL Matrigel™ (Corning, Corning, NY, USA) and 50 µL phosphate-buffered saline. After tumor engraftment (palpable tumors), tumor size was measured at least twice weekly using a digital caliper, and tumor volume was calculated using the formula V = (L × W²)/2. When tumors reached 5–7 mm in diameter, mice received 1 ×10^7^ CAR+ T cells in 100 µL phosphate-buffered saline via retro-orbital injection. Mice were sacrificed at day 7 or 10 after T cell transfer, and tumors were excised and processed using a previously described method (17). HLA-A*02 surface expression was subsequently analyzed as described above.

### Immunocompetent mouse model

Male and female A/J mice were subcutaneously injected with NXS2 tumor cells in 50 µL Matrigel™ and 50 µL phosphate-buffered saline. When tumors reached a size of 2–5 mm, 1 × 10^6^ GD2-CAR T cells were administered via retro-orbital injection. For the allogeneic setting, GD2-CAR T cells and untransduced T cells were generated from splenocytes of OT-I TCR transgenic mice (C57BL/6 background) carrying a homozygous *Rag2* knockout (*Rag2*^-^^/^^-^), while for the autologous setting, polyclonal CD3+ T cells from a A/J mouse were used. OT-I TCR transgenic T cells express a monoclonal TCR specific for ovalbumin, an antigen not expressed in mice and therefore do not induce graft-versus-host disease when transferred into mice of different genetic background. Seven days after T cell transfer, mice were sacrificed and tumors were excised and processed according to the protocol mentioned above. Cell surface MHC class I expression (H2-Dd) on NXS2 tumor cells was analyzed by flow cytometry.

### Statistical analysis

Data are expressed as mean ± standard error of the mean (SEM). Statistical analyses were performed using GraphPad Prism 10.1 (GraphPad Software, La Jolla, CA, USA). Data were analyzed using paired Student’s t-tests, two-way repeated-measures ANOVA, as indicated in the figure legends. The Geisser–Greenhouse correction was applied when the assumption of sphericity was violated. Mixed-effects models were used instead of repeated-measures ANOVA in cases of missing values. Multiple comparisons were performed using Šídák’s *post hoc* tests, as specified. Tests assuming equal variances were used where applicable. A *p*-value < 0.05 was considered statistically significant.

## Results

### IFNγ upregulates MHC class I in neuroblastoma cell lines

To explore the potential of combining TCR T cell therapy with CAR T cells in neuroblastoma, we first characterized MHC class I expression at baseline and after exposure to IFNγ across a panel of 27 neuroblastoma cell lines by staining for the classical MHC class I molecules HLA-A, HLA-B, and HLA-C (HLA-A,B,C). Flow cytometry revealed that many cell lines displayed very low or absent surface HLA-A,B,C expression, consistent with the well-established MHC class I-deficient phenotype of neuroblastoma (11, 25) (**Figure 1A**, **Supplementary Figure 1A**). Treatment with 0.1 ng/mL IFNγ for 24 h induced marked upregulation of HLA-A,B,C in most cell lines, in line with known IFNγ-dependent modulation of MHC class I expression in neuroblastoma. Notably, two cell lines, NB69 and SK-N-SH, failed to upregulate MHC class I upon IFNγ exposure, highlighting intrinsic resistance in a small subset of neuroblastoma models (**Figure 1A**). As many transgenic TCRs are restricted to HLA-A*02:01, we additionally assessed HLA-A*02 expression to identify neuroblastoma cell lines that could potentially be targeted by HLA-A*02:01-restricted TCR T cells. Among the nine identified HLA-A*02-positive cell lines (**Figure 1A**, **Supplementary Figure 1B**), LAN-1 cells displayed the lowest baseline HLA-A,B,C and HLA-A*02 expression but markedly upregulated both following IFNγ stimulation (**Figure 1A**, **Supplementary Figures 1A, B**). Parallel analysis of L1CAM expression (**Figure 1B**) identified LAN-1 suitable for targeting with L1CAM-CAR T cells (**Supplementary Figure 1C**). Based on these findings, LAN-1 cells were selected as a model system to investigate combinatorial targeting using L1CAM-CAR T cells and HLA-A*02:01 restricted TCR T cells.

**Figure 1:**
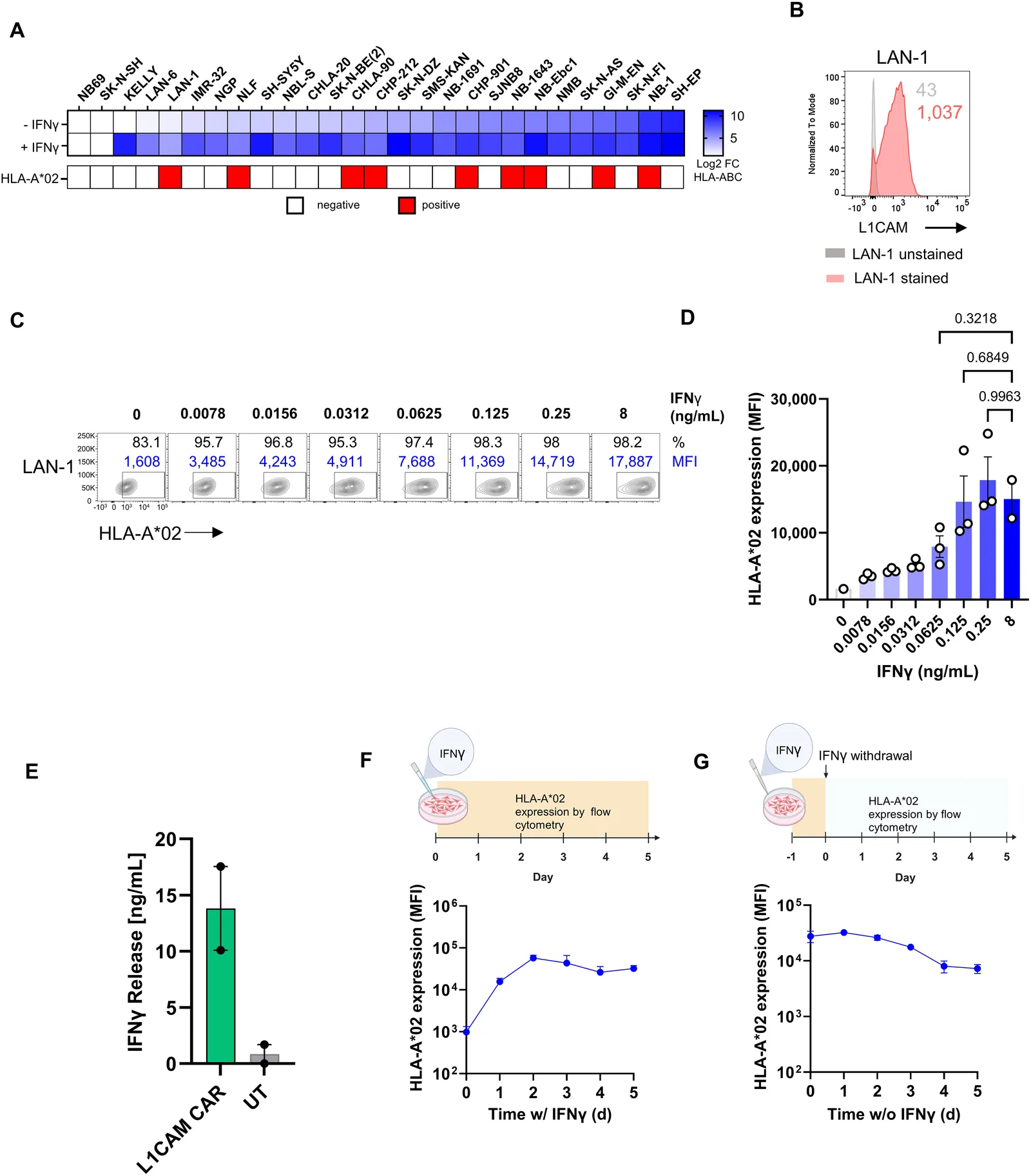
IFNγ upregulates MHC class I in neuroblastoma cell lines. **(A)** Flow cytometry-derived HLA-A,B,C surface expression, shown as log2 fold change (FC) in MFI after 24 h IFNγ treatment (0.1 ng/mL), in 27 neuroblastoma cell lines. Data are presented as a heatmap, with HLA-A*02 status indicated. Each cell line was analyzed in one to three independent experiments. **(B)** L1CAM expression on parental LAN-1 cells (red) was analyzed by flow cytometry. An unstained sample is shown for comparison (grey). Numbers within the blot indicate mean fluorescent intensity (MFI) values. Data are representative of three independent experiments. **(C)** Flow cytometry data showing the percentage of HLA-A*02–positive LAN-1 cells and magnitude of HLA-A*02 expression (expressed as MFI, blue) after 24 h exposure to indicated IFNγ concentrations (representative of n = 2–3 biological replicates from two independent experiments). **(D)** Summary of data described in **(C)**. Statistical significance was assessed using mixed effect analysis with Geisser-Greenhouse correction, followed by Šídák’s multiple-comparison test; only indicated comparisons were tested. Data are presented as mean ± SEM; n = 2–3 biological replicates obtained in two independent experiments. **(E)** IFNγ release of L1CAM-CAR T cells cocultured with LAN-1 cells for 24 h at a 1:1 E:T ratio, as determined with ELISA. Data are presented as mean ± SEM; n = 2 biological replicates from two independent experiments, using T cells from independent donors, with three technical replicates per biological replicate. No statistical analysis was performed due to the limited sample size. **(F)** HLA-A*02 upregulation in LAN-1 cells following exposure to a single IFNγ dose (10 ng/mL) administered at the start of the experiment, measured at indicated time points by flow cytometry; Data are presented as mean ± SEM; n = 2–3 biological replicates from two independent experiments. Created in BioRender. Kuenkele, A. (2026) https://BioRender.com/mlbelwh. **(G)** HLA-A*02 downregulation measured after incubation with 10 ng/mL IFNγ for 24 h (time point 0) followed by media change. (n = 2 independent experiments, each with technical replicates). Created in BioRender. Kuenkele, A. (2026) https://BioRender.com/mlbelwh.

To determine the minimal IFNγ concentration required to maximize MHC class I upregulation on LAN-1 cells, we incubated cells with titrated doses of IFNγ for 24 h and quantified HLA-A*02 expression by flow cytometry. A concentration of 0.25 ng/mL was sufficient to induce maximal HLA-A*02 expression (**Figures 1C, D**). Notably, this threshold is well below the levels of IFNγ secreted by L1CAM-CAR T cells during coculture with LAN-1 cells, where more than an order of magnitude higher concentration was detected (**Figure 1E**). These findings indicate that the amount of IFNγ produced by activated L1CAM-CAR T cells is sufficient to reach the concentration required for maximal HLA-A*02 induction.

Next, we assessed the kinetics of HLA-A*02 upregulation following a single dose of IFNγ (10 ng/mL), administered once at the start of the experiment, and monitored HLA-A*02 expression daily over 5 days by flow cytometry (**Figure 1F**). HLA-A*02 expression in LAN-1 cells peaked on day 2 and remained stable over the remaining time of the analyzing period. To evaluate the persistence of MHC class I expression, IFNγ was removed at peak induction, and HLA-A*02 levels were subsequently tracked by flow cytometry. Notably, expression remained elevated for at least 5 days after cytokine withdrawal (**Figure 1G**), suggesting that, under the conditions tested, tumor cells may remain susceptible to TCR T cell targeting even in the absence of continuous IFNγ exposure.

Collectively, these findings are consistent with established evidence that IFNγ efficiently restores MHC class I expression in the majority of neuroblastoma models. Importantly, the levels of IFNγ produced by activated L1CAM-CAR T cells exceeded the threshold required for maximal HLA-A*02 induction, supporting the rationale that CAR T cell-derived IFNγ may sensitize neuroblastoma cells to subsequent antigen-specific TCR T cell recognition and killing.

### IFNγ incubation recovers activity of NY-ESO-1 TCR T cells

We then assessed whether the sustained IFNγ-induced MHC class I upregulation enables effective recognition and killing by TCR-engineered T cells. We focused on NY-ESO-1, an attractive antigen for TCR T cell therapy. Although this antigen was detected in only a small subset of neuroblastoma patient samples (**Supplementary Figure 2A**), it was readily detectable and expressed at appreciable levels in LAN-1 cells (**Supplementary Figures 2B, C**). Thus, NY-ESO-1-directed TCR T cell therapy was primarily employed here as a proof-of-concept model for antigen-specific TCR T cell therapy, rather than to specifically investigate NY-ESO-1 as a therapeutic target in NY-ESO-1-positive neuroblastoma.

We employed a transgenic, high-affinity NY-ESO-1-specific TCR derived from a human TCR-transgenic mouse model and restricted to HLA-A*02:01 (16). As this receptor contains murine TCR constant regions, transduced T cells could be readily identified and enriched by MACS using an antibody specific for the murine TCR β constant region (TRBC; **Supplementary Figures 2E, F**). The mean NY-ESO-1 TCR expression increased from 22.4% ± 13.04% before MACS sorting to 74.7% ± 16.9% after sorting (mean ± SEM, n = 5), demonstrating effective enrichment of NY-ESO-1 TCR-expressing cells. Real-time cytotoxicity assays using the IncuCyte platform revealed that NY-ESO-1 transgenic TCR T cells exhibited minimal killing of untreated LAN-1 tumor cells over 72 h (**Figures 2A, B**). In contrast, pretreatment of LAN-1 cells with IFNγ markedly enhanced susceptibility to NY-ESO-1 TCR T cell-mediated cytotoxicity, resulting in a 92.8% ± 0.34 (mean ± SEM; n = 3) reduction of tumor cell expansion compared to tumor cells treated with NY-ESO-1 TCR T cells alone (p = 0.0597). This reduction was comparable to that observed following IFNγ pretreatment combined with exogenous NY-ESO-1 peptide pulsing prior to NY-ESO-1 TCR T cell treatment, which resulted in a 91.5% ± 1.93% reduction in tumor cell expansion (mean ± SEM; n = 3). Although the specific HLA-A*02 subtype expressed by LAN-1 was not formally determined, the robust activation and antitumor response of NY-ESO-1 TCR T cells following induction of HLA-A*02 expression strongly support that LAN-1 cells express HLA-A*02:01 or another HLA-A*02 subtype capable of presenting the NY-ESO-1_157__-__165_ epitope recognized by the used TCR (17). Importantly, untransduced T cells failed to induce relevant tumor cell killing irrespective of IFNγ pretreatment or NY-ESO-1 peptide pulsing, confirming antigen- and TCR-dependent cytotoxicity. Consistent with these findings, NY-ESO-1 TCR T cells stimulated with IFNγ-pretreated LAN-1 cells secreted higher levels of IFNγ and IL-2 (**Figure 2C**) and more frequently co-expressed the activation markers CD25, CD137, and CD69 (**Figure 2D**) compared with T cells stimulated with untreated LAN-1 neuroblastoma cells, although these differences did not reach statistical significance. Together, these findings suggests that, in a representative neuroblastoma cell line, low baseline MHC class I expression contributes to limited recognition and killing of neuroblastoma cells by NY-ESO-1 TCR T cells, whereas exposure to IFNγ at physiological concentrations increases tumor susceptibility to TCR T cell-mediated cytotoxicity.

**Figure 2:**
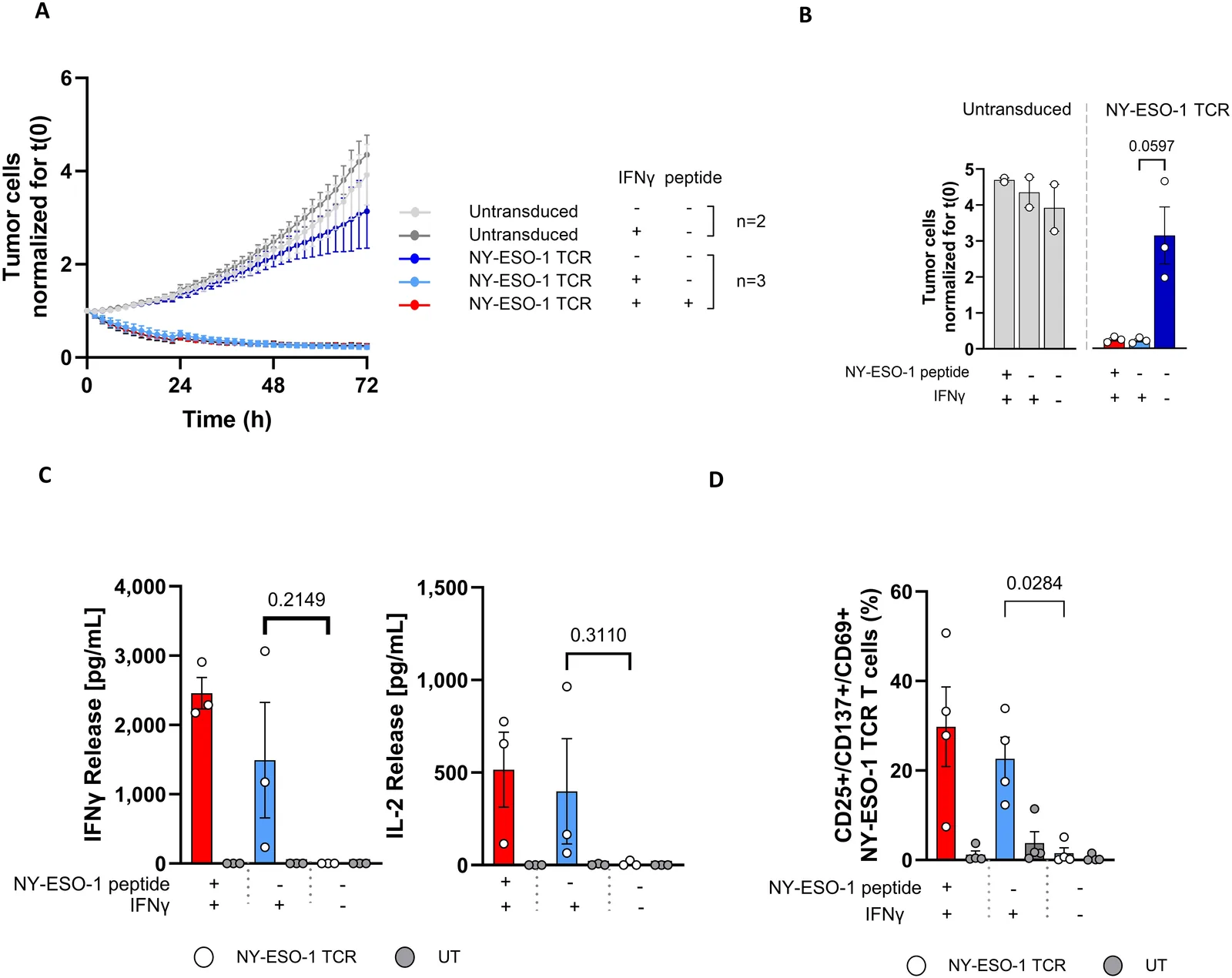
Low MHC class I expression in neuroblastoma results in inadequate recognition and killing by NY-ESO-1 TCR T cells. **(A)** 72-hour IncuCyte killing assay of untransduced or NY-ESO-1 TCR T cells cocultured with parental LAN-1 neuroblastoma cells, with or without IFNγ pretreatment (10 ng/mL) or NY-ESO-1 peptide pulsing (10 nM), at an E:T ratio of 1:1 (n = 2–3 biological replicates, each performed with technical triplicates). **(B)** Tumor cell counts from the cocultures shown in **(A)** after 72 h, normalized to baseline (0 h). **(C)** Cytokine release and **(D)** CD137+/CD25+/CD69+ triple surface expression of untransduced and NY-ESO-1 TCR T cells cocultured with parental LAN-1 with or without IFNγ pretreatment (10 ng/mL) or peptide pulsing (10 nM) at 1:1 E:T ratio (n = 3–4 biological replicates using independent T cell donors, each with technical triplicates). Data are expressed as mean ± SEM. Statistical significance in **(B–D)** was analyzed using a paired Student’s t-test.

### L1CAM-CAR T cell treatment primes neuroblastoma cells and restores NY-ESO-1 TCR T cells activity

Next, we investigated whether pretreatment with L1CAM-CAR T cells could functionally prime tumor cells in a manner comparable to direct IFNγ stimulation. To better approximate a clinically relevant two-product setting, we envisioned generating the CAR T cell product from healthy donor T cells. To avoid graft-versus-host reactions, the L1CAM-CAR was knocked into the *TRAC* locus using CRISPR/Cas9 that results in simultaneous knockout of the endogenous TCR, as demonstrated by the loss of cell surface CD3 expression (**Supplementary Figure 3A**). L1CAM-CAR knock-in did not significantly alter the CD4/CD8 ratio of the CAR T cell product compared with unmodified T cells (p = 0.076; **Supplementary Figure 3B**). We also generated a LAN-1 cell line in which L1CAM was disrupted (LAN-1 L1CAM KO) using CRISPR/Cas9, which served as a negative control. Successful knockout of L1CAM was confirmed by flow cytometry (**Supplementary Figure 3C**). L1CAM-deficient LAN-1 cells showed comparable HLA-A*02 expression at baseline and following stimulation with 10 ng/mL IFNγ (**Supplementary Figure 3D**). Although L1CAM knockout cells exhibited an initial lag phase, they subsequently proliferated similarly to parental LAN-1 cells (p=0.3052; **Supplementary Figure 3E**). Moreover, IFNγ treatment did not affect cell viability (**Supplementary Figure 3F**) or growth kinetics (**Supplementary Figure 3G**), nor did it alter L1CAM or NY-ESO-1 expression levels in either parental of L1CAM KO LAN-1 cells (**Supplementary Figures 3H, I**).

Parental LAN-1 or LAN-1 L1CAM KO were cocultured *in vitro* with L1CAM-specific CAR T cells for 24 h at a deliberately suboptimal E:T ratio to achieve incomplete tumor cell killing while allowing CAR T cell-mediated priming. NY-ESO-1 TCR T cells were then added 24 h later (**Figure 3A**). This 24-h priming interval was selected to allow sufficient CAR T cell activation and IFNγ-mediated tumor cell priming before TCR T cell addition. Following coculture, cytotoxicity against LAN-1 and LAN-1 L1CAM KO was assessed, along with NY-ESO-1 TCR T cell activation, IFNγ and IL-2 release, and HLA-A*02 expression on parental LAN-1 cells at indicated time points. Compared with untreated tumor cells, monotherapy with either L1CAM-CAR T or NY-ESO-1 TCR T cells showed only modest cytotoxic activity against parental LAN-1 cells, resulting in a 15.0% ± 32.5% (mean ± SEM; n = 3) and 14.2% ± 3.2% (mean ± SEM; n = 3) reduction in tumor cell expansion, respectively. As expected, treatment of LAN-1 L1CAM KO cells resulted in no (−16.8% ± 6.5%; mean ± SEM; n = 3) and 28.3% ± 9.0% (mean ± SEM; n = 3) reduction following L1CAM-CAR T and NY-ESO-1 TCR T cell monotherapy, respectively (**Figures 3B, C**). In the combined T cell setting, pretreatment of L1CAM-positive LAN-1 cells with L1CAM-CAR T cells enhanced the functional activity of NY-ESO-1 TCR T cells, resulting in a 76.5% ± 14.7% reduction in tumor cell expansion (mean ± SEM; n = 3) compared with untreated cells (p = 0.2458), recapitulating the effect observed with IFNγ pretreatment (**Figure 2B**). Cytotoxicity was less pronounced in LAN-1 L1CAM KO cells, with a 48.7% ± 17.6% reduction in cell growth (mean ± SEM; n = 3; p = 0.3603), consistent with a requirement for L1CAM-dependent CAR T cell activation to maximize subsequent TCR-mediated cytotoxicity (**Figures 3B, C**). Differences in tumor cell killing between conditions with or without CAR T cell pretreatment did not reach statistical significance. Flow cytometry analysis revealed that NY-ESO-1 TCR T cells expressed the activation markers CD25 and CD137 at higher frequency in the combined treatment setting (a detailed gating strategy is shown in **Supplementary Figure 3J**). In contrast, CD25 expression was reduced and CD137 was nearly absent on NY-ESO-1 T cells under monotherapy conditions (**Figure 3D**). Similarly, IFNγ and IL-2 levels detected in cocultures with combined T cell treatment were elevated, similarly as in cocultures with IFNγ stimulated LAN-1 cells, while the release of both cytokines remained under the detection limit in cocultures with NY-ESO-1 TCR treatment alone (**Figures 3E, F**). In line with these findings, NY-ESO-1 T cell monotherapy (TCR) did not elevate the HLA-A2 expression in parental LAN-1 cells above baseline levels (−IFNγ, **Figure 3G**), whereas combined T cell treatment (CAR + TCR) resulted in robust upregulation of MHC class I expression, reaching levels comparable to those observed following IFNγ stimulation or L1CAM-CAR T cell treatment. Collectively, these data demonstrate that tumor cell-mediated activation of L1CAM-CAR T cells, results in the release of sufficient amounts of effector molecules, including IFNγ and other proinflammatory factors capable of upregulating MHC class expression. These factors functionally prime neuroblastoma cells, thereby enhancing their recognition and cytotoxicity by NY-ESO-1 TCR T cells. This effect mimics the enhanced tumor cell recognition overserved following IFNγ-induced MHC class I upregulation.

**Figure 3:**
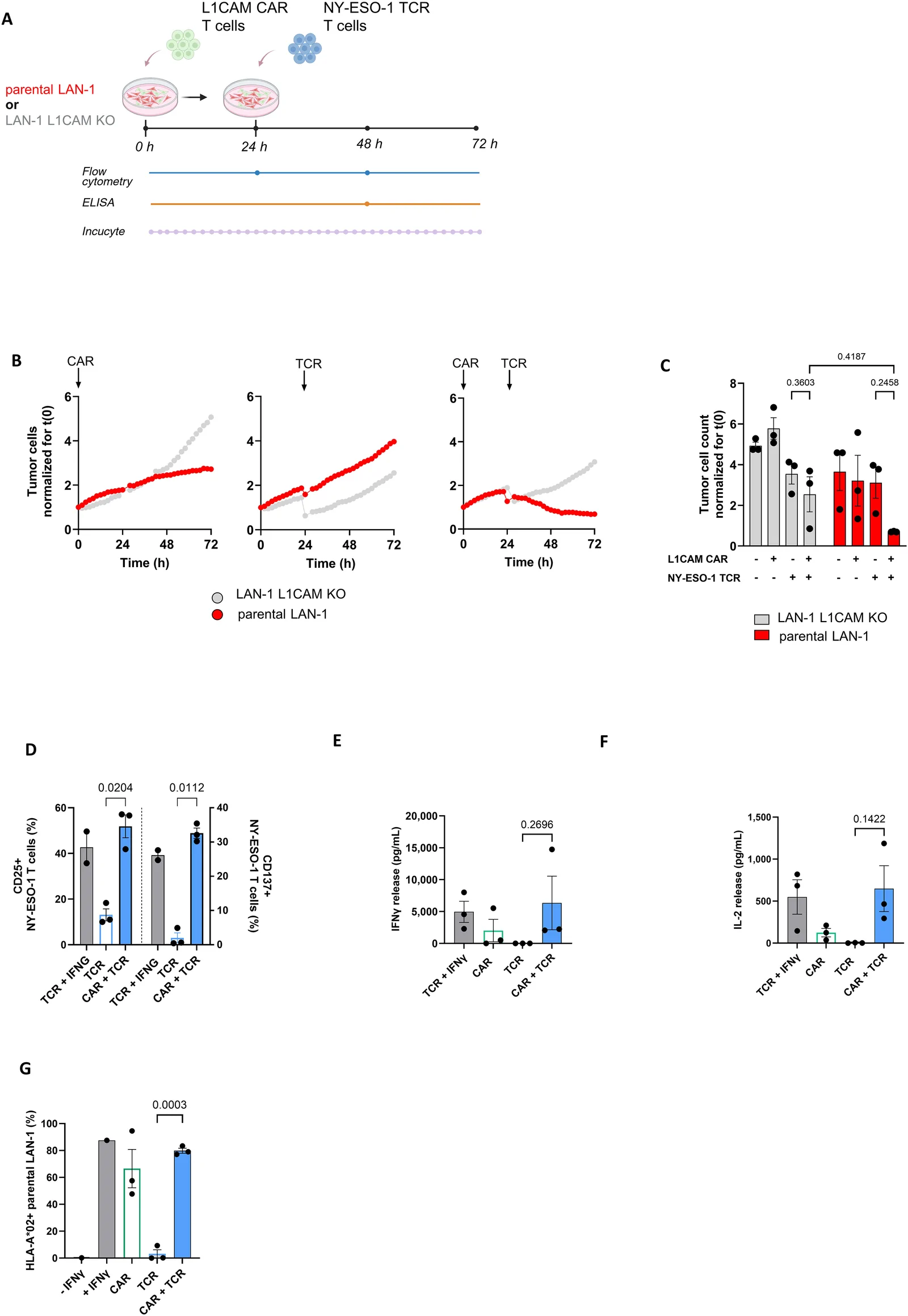
L1CAM-CAR T cells pre-treatment induces MHC class I upregulation and rescues NY-ESO-1 TCR T cell cytotoxicity against neuroblastoma cells. **(A)** Schematic of the experimental setup. L1CAM-CAR T cells were cocultured for 24 h with parental LAN-1 or LAN-1 L1CAM KO target cells in an E:T ratio of 1:1. Subsequently, the same number of NY-ESO-1 TCR T cells were added. Killing kinetics were monitored over 72 h using IncuCyte measurements. In parallel, activation marker expression on NY-ESO-1 TCR T cells and HLA-A*02 expression on LAN-1 cells were assessed by flow cytometry, and IFNγ and IL-2 secretion was quantified by ELISA. Created in BioRender. Kuenkele, A. (2026) https://BioRender.com/mlbelwh. **(B)** Representative IncuCyte-based killing kinetics from a single experiment showing L1CAM-CAR T cells, NY-ESO-1 TCR T cells, and their combination (1:1 ratio) cocultured with parental LAN-1 (red) or LAN-1 L1CAM KO (grey) target cells. **(C)** Tumor cell counts of cocultures shown in **(B)** after 72 h normalized to baseline (0 h). Statistical significance was assessed using a two-way repeated-measures ANOVA with Geisser-Greenhouse correction, followed by Šídák’s multiple-comparison test; only indicated comparisons were tested. **(D)** CD25 and CD137 expression on NY-ESO-1 TCR T cells cocultured with parental LAN-1 cells. **(E)** IFNγ and **(F)** IL-2 release measured 48 h after initiation of coculture. **(G)** Bar graph depicting HLA-A*02 expression on the surface of parental LAN-1 cells. Statistical significance in panel **(D–G)** was assessed using a paired Student’s t-test. Data in **(C–G)** are presented as mean ± SEM; n = 2–3 biological replicates from 2–3 independent experiments using T cells from independent donors.

### L1CAM-CAR pretreatment restored NY-ESO-1 TCR T cell therapy despite heterogeneous L1CAM expression

Heterogeneous antigen expression represents a major challenge for targeted immunotherapies by promoting the emergence of therapy-resistant tumor variants and subsequent tumor recurrence. To test whether our dual approach can not only restore NY-ESO-1 TCR T cell activity but also effectively target a tumor population with heterogeneous antigen expression, we established a model that simulates antigen heterogeneity, by mixing LAN-1 and LAN-1 L1CAM KO in a 1:1 ratio. To enable discrimination between both tumor cell populations, parental LAN-1 and LAN-1 L1CAM KO cells were lentivirally transduced to express either a red or green fluorescent protein fused to a nuclear localization signal (NLS-mKate or NLS-GFP), respectively (**Supplementary Figure 4A**). As before, the LAN-1 cell mixture was first stimulated with L1CAM-CAR T cells for 24 h before the addition of NY-ESO-1 TCR T cells (**Figure 4A**). When cocultured with the mixed tumor cell population, L1CAM-CAR T cells showed modest cytotoxic activity against parental LAN-1 cells, resulting in a 13.5% ± 10.6% reduction in tumor cell expansion compared with untreated cells (mean ± SEM; n = 3; **Figures 4B, C**). In contrast no cytotoxic activity was observed against LAN-1 L1CAM KO cells, which showed no change in tumor cell expansion (−28.9% ± 5.0%; mean ± SEM; n = 3). Similarly, NY-ESO-1 TCR T cells alone showed no relevant cytotoxic activity against either tumor cell population, resulting in a 1.4% ± 15.9% (mean ± SEM; n = 3) and 12.1% ± 15.2% (mean ± SEM; n = 3) reduction in tumor cell expansion in parental LAN-1 and LAN-1 L1CAM KO cells, respectively. However, when NY-ESO-1 TCR T cells were added to the tumor cell mix following pretreatment with L1CAM-CAR T cells, TCR T cell-mediated cytotoxicity was markedly enhanced, resulting in a 59.8% ± 10.6% reduction in tumor cell expansion in parental LAN-1 cells and an 87.4% ± 9.8% reduction in LAN-1 L1CAM KO cells (mean ± SEM; n = 3). This effect was accompanied by increased NY-ESO-1 TCR T cells activation, characterized by a significantly higher proportion of CD25- and CD137-positive T cells (**Figure 4D**). The detailed gating strategy used to distinguish individual T cell populations from tumor cells is shown in **Supplementary Figure 4B**. This enhanced activation was further supported by increased release of IFNγ (**Figure 4E**) and IL-2 (**Figure 4F**) compared to the NY-ESO-1 TCR T cell monotherapy. Importantly, we observed a significant upregulation of HLA-A*02 on the LAN-1 L1CAM KO population in the combined T cell treatment condition (**Figures 4G, H**), which likely enhanced NY-ESO-1 peptide presentation and facilitated subsequent NY-ESO-1 TCR T cell-mediated killing. This demonstrated that CAR antigen expression was not required on all tumor cells, as IFNγ released by CAR T cells primed both L1CAM-expressing and L1CAM-deficient tumor cells for subsequent NY-ESO-1 TCR T cell-mediated killing. Together, these findings demonstrate that sequential CAR and TCR T cell therapy can not only render MHC class I-low neuroblastoma cells susceptible to efficient TCR-mediated killing, but also overcome tumor antigen heterogeneity by converting initially resistant, CAR antigen-negative tumor cells into targets for TCR-mediated elimination.

**Figure 4:**
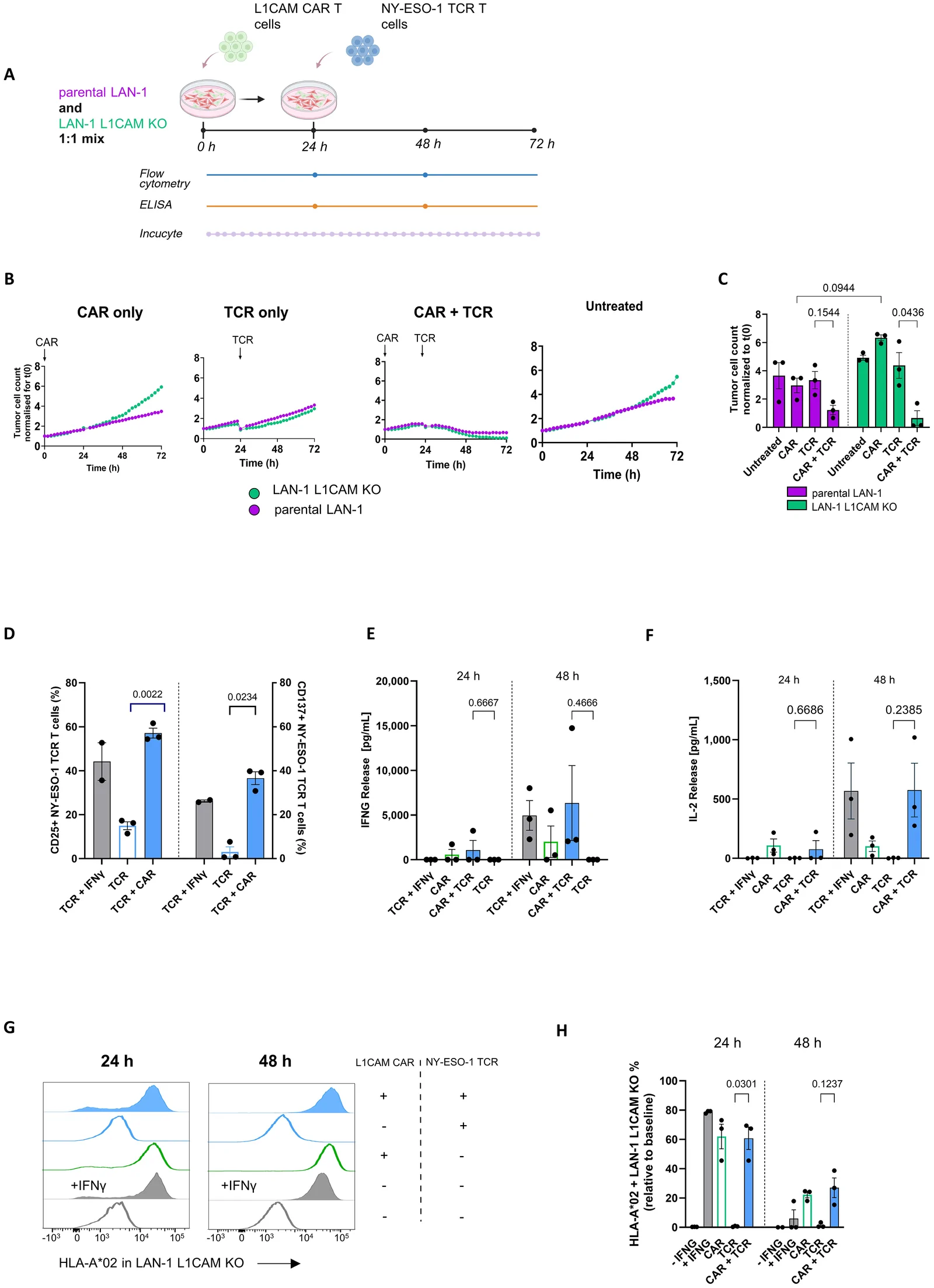
Neuroblastoma cells with heterogeneous CAR antigen expression uniformly upregulate MHC class I following CAR T cell pre-treatment and become sensitized to NY-ESO-1 TCR T cell attack. **(A)** Experimental scheme illustrating the coculture setup. Parental LAN-1 (magenta) and LAN-1 L1CAM KO (green) target cells were mixed 1:1 and cocultured first with L1CAM-CAR T cells (E:T ratio 1:1). After 24 h, the same amount of NY-ESO 1 TCR T cells were added. Created in BioRender. Kuenkele, A. (2026) https://BioRender.com/mlbelwh **(B)** Representative IncuCyte-based real-time cytotoxicity curves showing the number of parental LAN-1 (magenta) and LAN-1 L1CAM KO (green) tumor cells following treatment with L1CAM-CAR T cells (left panel), NY-ESO-1 TCR T cells (middle panel), and their combination (right panel). Growth of untreated tumor cells is shown as control. **(C)** Quantification of the 72-h endpoint from the corresponding IncuCyte assays shown in **(B)**, summarizing data from three independent IncuCyte-based killing assays. Statistical significance was assessed using a two-way repeated-measures ANOVA with Geisser-Greenhouse correction, followed by Šídák’s multiple-comparison test; only indicated comparisons were tested. **(D)** CD25 and CD137 expression on NY-ESO-1 TCR T cells cultured with parental LAN-1 and LAN-1 L1CAM KO 1:1 mix alone or in combination with L1CAM-CAR T cells. The condition in which LAN-1 cell mix was pretreated with IFNγ before addition of T cells served as positive control. Statistical significance was assessed using paired, two-tailed t-test; only the indicated comparisons were tested. **(E)** IFNγ and **(F)** IL-2 release of L1CAM-CAR and/or NY-ESO-1 TCR T cells after 24 h or 48 h after coculture initiation. **(G)** Exemplary histograms and **(H)** summary of flow cytometry analysis showing HLA-A*02 expression on the surface of LAN-1 L1CAM KO cells after indicated treatments. A detailed gating strategy is shown in **Supplementary Figure 4B**. Statistical significance was assessed using a two-way repeated-measures ANOVA with Geisser-Greenhouse correction, followed by Šídák’s multiple-comparison test; only indicated comparisons were tested. Data in **(C–H)** are presented as mean ± SEM; n = 3 biological replicates from 3 independent experiments using T cells from independent donors.

### L1CAM-CAR T cells upregulate HLA-A*02 *in vivo*

To determine whether the CAR T cell-mediated HLA upregulation observed *in vitro* could also be induced *in vivo*, and to define the magnitude and kinetics of this response, we next evaluated MHC class I expression following L1CAM-CAR T cell therapy in a xenograft model. As outlined in **Figure 5A**, mice were engrafted subcutaneously with LAN-1 cells and treated with L1CAM-CAR T cells upon establishment of palpable tumors (5 to 7 mm in diameter). Of note, due to the relative low transduction efficacy, L1CAM CAR T cells required enrichment by MACS and extensive expansion using a rapid expansion protocol (REP; see Methods) to obtain sufficient cell numbers. Although exhaustion marker expression remained modest directly after the REP compared with cells stimulated three times with tumor cells (**Supplementary Figure 5A**), the REP induced a more differentiated T cell phenotype than in non-REP T cells, accompanied by an increase in the effector memory T cell population from 18.4% to 48.7%. (**Supplementary Figure 5B**). Tumors were harvested on day 7 and 10 post-treatment and analyzed via flow cytometry. At day 7, tumors from CAR T cell-treated mice exhibited a detectable increase in HLA-A*02 expression level compared with untreated controls (**Figures 5B, C**). However, this increase did not reach statistical significance. Interestingly, tumors treated with untransduced T cells also exhibited a slight HLA-A*02 increase, which may reflect low-level allogeneic/xenogenic responses in this setting. HLA-A*02 upregulation, however, was more pronounced following exposure to L1CAM-specific CAR T cells (mean MFI ± SEM, L1CAM-CAR vs untransduced CAR: 415 ± 65, n =3 vs 314 ± 31, n =3), supporting an L1CAM-dependent component of CAR T cell-mediated priming. HLA-A*02 expression analysis on day 10 showed no upregulation in any group, indicating that the peak of HLA-A*02 upregulation was reached earlier. These results demonstrate that L1CAM-CAR T cell therapy can enhance HLA-A*02 expression *in vivo*, with peak induction occurring at or before day 7. However, HLA-A*02 upregulation was modest and transient, which may be related to the more differentiated phenotype of L1CAM-CAR T cells after extensive *in vitro* expansion during REP.

**Figure 5:**
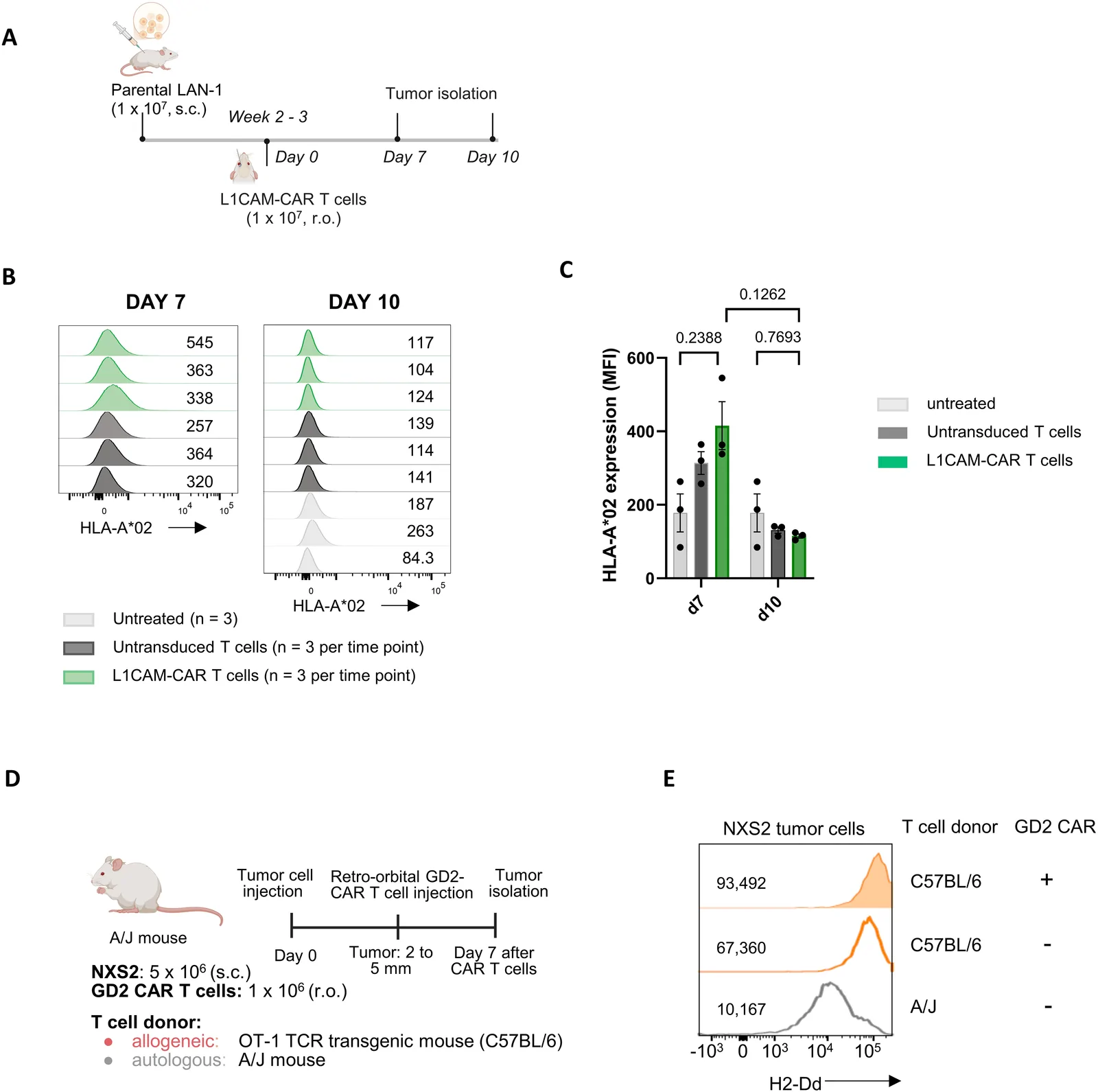
L1CAM-CAR T cells induce HLA-A*02 upregulation on neuroblastoma cells *in vivo*. **(A)** Schematic overview of experimental workflow. NSG mice were subcutaneously inoculated with 1 x 10^7^ parental LAN-1 cells. When the tumor reached a size of 5 mm, mice were treated with 1 x 10^7^ L1CAM-CAR T cells applied retro-orbitally (r.o.). Tumors were isolated on day 7 or day 10 after T cell application to analyze HLA-A*02 expression. Created in BioRender. Kuenkele, A. (2026) https://BioRender.com/mlbelwh. **(B)** Histograms of tumor cells isolated from individual NSG mice and **(C)** quantification of the corresponding flow cytometry analysis showing HLA-A*02 expression on parental LAN-1 tumor cells isolated from NSG mice on day 7 or 10 after L1CAM-CAR T cell treatment. Numbers in **(B)** indicate mean fluorescent intensity (MFI) values. Statistical significance was assessed using a two-way repeated-measures ANOVA with Geisser-Greenhouse correction, followed by Šídák’s multiple-comparison test; only indicated comparisons were tested. -**(D)** Experimental setup. A/J mice were subcutaneously inoculated with 5 x 10^6^ NXS2 cells. When tumors reached a size of max 5 mm in diameter, mice were treated with 1 x 10^6^ GD2-CAR T cells derived from a C57BL/6 T cell donor mouse (allogeneic setting). Untransduced T cells from an A/J or C57BL/6 donor mouse served as controls. Tumors were isolated seven days after GD2-CAR T cell treatment (n = 1 per group) and MHC class I (H2-Dd) expression on NXS2 cells was analyzed by flow cytometry. **(E)** Histograms show *ex vivo* H2-Dd expression on NXS2 tumor cells seven days after indicated T cell treatment. Numbers indicate MFI (n = 1).

Next, we evaluated whether an allogeneic CAR T cell product could be used in a clinically relevant two-T cell-product scenario. To minimize genetic engineering of the allogeneic CAR T cells, the CAR would be knocked in into the *TRAC* locus, thereby eliminating endogenous TCR expression and preventing graft-versus-host reactions. However, because MHC expression is retained, these CAR T cells remain susceptible to recognition and rejection by the host immune system. To determine whether the resulting window of CAR T cell persistence would be sufficient to enhance tumor MHC class I expression, we evaluated this an immunocompetent mouse model. Mice bearing NXS2 tumors were treated with either autologous GD2-CAR T cells or an allogeneic GD2-CAR T cell product derived from an OT-I TCR transgenic mouse (**Figure 5D**). We used GD2-directed CAR T cells (**Supplementary Figure 5C**), as GD2 is conserved between humans and mice. Seven days after treatment, tumors were excised and analyzed. Notably, tumors were small in most mice, and viable tumor cells could be recovered from only one mouse per group (1/3). MHC class I (H2-Dd) expression was increased on tumor cells isolated from both mice treated with allogeneic T cells compared with those treated with autologous untransduced T cells, independent of GD2-CAR expression (**Figure 5E**). These findings suggest that the allogeneic CAR T cells persisted sufficiently to reach the tumor and induce IFNγ-mediated MHC upregulation. The observation that this effect was also present in the mouse treated with allogeneic T cells lacking the GD2-CAR may reflect an alloreactive host T cell response, contributing to IFNγ production. Given the exploratory nature and limited sample size, these findings should be interpreted cautiously and considered preliminary evidence that minimally engineered allogeneic T cells may modulate the tumor microenvironment and antigen presentation. Further studies with larger cohorts are warranted.

## Discussion

TCR-mediated T cell therapy represents a promising therapeutic strategy for refractory and high-risk neuroblastoma; however, low or absent MHC class I expression constitutes a major barrier to its efficacy. In this study, we demonstrate that CAR T cells can serve as an effective source of inflammatory signaling to upregulate MHC class I expression in neuroblastoma, thereby sensitizing tumor cells to subsequent TCR-mediated T cell therapy. Thus, CAR T cells act not only as direct effector cells, but also as “priming agents” that enable subsequent eradication of tumor cells by TCR-engineered T cells. A similar strategy has been described, in which NK cell-mediated “missing-self” recognition was harnessed to induce an IFNγ-response, thereby enhancing subsequent TCR-mediated T cell reactivity against neuroblastoma (26). While this study established that IFNγ-mediated inflammatory signaling can overcome the MHC class I-low phenotype of neuroblastoma, our findings extend this concept to CAR T cell therapy derived from healthy donors. The use of a healthy-donor-derived CAR T cell product may further enhance the translational feasibility of this strategy. Rather than requiring individualized manufacture of two cellular products from the same pediatric patient, a standardized allogeneic CAR T cell product could be manufactured in advance, potentially reducing manufacturing complexity. This may be particularly relevant in the pediatric setting, where limited blood volumes, the availability and quality of patient-derived starting material, and the feasibility of repeated or complex leukapheresis procedures can pose important practical constraints. While allogeneic CAR T cells may themselves be recognized by the host immune system, triggering IFNγ release, this immune recognition could potentially contribute to the priming effect by further enhancing IFNγ-mediated upregulation of HLA-A,B,C on tumor cells. At the same time, transient persistence of the allogeneic CAR T cells due to their immune-mediated clearance may limit prolonged systemic cytokine levels, reducing the risk of cytokine toxicity potentially associated with administering two T cell products. In addition, engineering of neuroblastoma cells using CRISPR/Cas9-mediated *IFNG* gene knock-in, exploiting novel PAM sites generated by tumor-specific mutations, provides another example of an approach to enhance MHC class I expression in neuroblastoma (27). In this approach, tumor cells were directly engineered to produce IFNγ, resulting in enhanced MHC class I expression. Although this strategy similarly exploits IFNγ-mediated remodeling of the neuroblastoma phenotype, it relies on efficient *in vivo* genetic modification of the tumor cells themselves to establish IFNγ production.

We demonstrated across a panel of 27 neuroblastoma cell lines that basal MHC class I expression was low in the majority of models, but could be readily restored following IFNγ stimulation *in vitro*. The low MHC class I phenotype in neuroblastoma is thought to be linked to its embryonic origin. Neuroblastoma cells derive from neural crest-related precursor cells that are arrested at an early stage of differentiation. While NF-κB signaling plays a key role in the maturation of neural crest precursor cells (28), the observed lack of NF-κB activity in neuroblastoma cells (29) are in line with its maintenance in an undifferentiated state. Importantly, NF-κB directly regulates the transcription of MHC class I heavy chain genes as well as β2-microglobulin (30), explaining mechanistically the MHC-low phenotype. Consistently, a genome-wide CRISPR screen identified N4BP1 and TNIP1 as key inhibitory regulators of NF-κB signaling in neuroblastoma, and their deletion resulted in increased MHC class I expression in neuroblastoma cell lines (30). Thus, therapeutic strategies aimed at restoring NF-κB activity may also enhance MHC class I expression and sensitize neuroblastoma to TCR-mediated T cell therapy. However, such approaches would not exploit the additional advantage of combinatorial CAR/TCR strategies, which can simultaneously address tumor immune evasion driven by antigen loss and heterogeneity. However, restoration of MHC class I expression may be largely restricted to embryonal tumors, in which antigen presentation is suppressed as part of a developmentally regulated cellular state. In contrast, MHC class I deficiency in many adult cancers is frequently caused by more stable genetic alterations, most commonly involving deletion or mutations in the *B2M* gene (31) or defects in components of the antigen-processing machinery (32). Interestingly, two of the neuroblastoma cell lines analyzed in our study failed to upregulate MHC class I following IFNγ stimulation, suggesting that they may harbor defects affecting the IFNγ-MHC class I axis analogous to those found in adult cancer. We did not investigate the underlying mechanism in these two cell lines further, but their IFNγ-nonresponsive phenotype suggests that, in a small subset of neuroblastomas, MHC class I deficiency may involve mechanisms beyond the developmentally regulated suppression of antigen presentation.

To experimentally evaluate a combinatorial CAR/TCR strategy in neuroblastoma, we established a model using L1CAM-targeting CAR T cells in combination with NY-ESO-1-specific TCR-engineered T cells in the LAN-1 neuroblastoma cell line. Although NY-ESO-1 protein expression has been reported in 28%-36% of neuroblastoma samples by RT-PCR and immunohistochemistry (7, 11) our analysis of publicly available RNA-sequencing datasets from neuroblastoma patients suggested that robust NY-ESO-1 transcription is confined to a relatively small subset of patients. Nonetheless, prior studies have shown that several cancer-testis antigens, including NY-ESO-1, can be upregulated following exposure to pharmacologic doses of the demethylating agent 5-aza-2′-deoxycytidine (decitabine, DAC) (33). Moreover, combining demethylating chemotherapy with cancer antigen vaccination has resulted in complete remission of persistent tumor cells in the bone marrow, demonstrated in a single reported patient (34). At the same time, several alternative TCR targets with broader expression profiles, including PRAME (35), represent promising candidates, and ongoing antigen discovery efforts are likely to expand the repertoire of suitable intracellular targets for combinatorial CAR/TCR approaches in neuroblastoma.

The limited and heterogeneous expression of target antigens highlights a fundamental challenge in neuroblastoma immunotherapy. The intrinsic plasticity of neuroblastoma, which enables transitions between distinct cellular states characterized by differential gene expression profiles, including therapeutic target antigens, makes this tumor particularly prone to immune escape following targeted therapy. Previous studies demonstrated that transition from a more differentiated adrenergic state to a less differentiated mesenchymal-like state is associated with loss of GD2 expression, resulting in resistance to GD2-targeting antibody therapy (13). Consistently, we previously observed that induction of oncogenic MYCN expression, the key oncogenic driver in neuroblastoma, led to downregulation of L1CAM, another clinically relevant CAR target antigen (36). In contrast, pharmacological inhibition of ALK, another important oncogenic driver in a subset of neuroblastomas, resulted in upregulation of ALK and enhanced susceptibility to ALK-targeting CAR T cell therapy (37). Together, these findings highlight the dynamic regulation of antigen expression in neuroblastoma in response to oncogenic signaling and therapeutic pressure, and underscore that careful selection and strategic combination of target antigens may be required to achieve durable tumor control in this highly plastic malignancy.

This study has several limitations. First, enhanced NY-ESO-1 TCR T cell-mediated killing following IFNγ-induced MHC class I upregulation was functionally demonstrated in a single neuroblastoma cell line. Validation in additional HLA-A*02:01-positive, NY-ESO-1-expressing neuroblastoma models will be important to further establish the generalizability of this approach. Second, we demonstrated that maximal HLA-A*02 expression could be induced with a relatively low concentration of IFNγ, while L1CAM-CAR T cells stimulated with LAN-1 cells at an E:T of 1:1 produced IFNγ at levels more than an order of magnitude higher than those required for maximal HLA-A*02 induction. Nevertheless, the induction of MHC class I expression *in vivo* remained modest and transient, which may reflect the limited functional fitness of the extensively expanded CAR T cells used in this study. Future efforts should focus on manufacturing strategies that improve CAR T cell quality and persistence to enhance their capacity for tumor modulation. Finally, while our data demonstrate that CAR T cell-mediated inflammatory signaling can sensitize tumor cells to subsequent TCR T cell recognition *in vitro*, sequential CAR T and TCR T treatment was not evaluated *in vivo*. Further studies directly comparing CAR T cell monotherapy, TCR T cell monotherapy, and sequential CAR T/TCR T treatment will be required to define the therapeutic potential and optimal implementation of this combinatorial strategy.

In summary, our findings support a sequential immunotherapy concept in which CAR T cells function as an upstream inflammatory stimulus to enhance MHC class I expression and potentially increase the susceptibility of neuroblastoma cells to subsequent targeting by TCR-engineered T cells. This approach provides a potential strategy to address the limited antigen presentation that can restrict TCR-mediated recognition in neuroblastoma and illustrates how complementary T cell modalities may be combined to enhance anti-tumor responses. Further studies will be required to determine the *in vivo* relevance, and therapeutic potential of this approach.

## Data Availability

The original contributions presented in the study are included in the article/**Supplementary Material**. Further inquiries can be directed to the corresponding author.

## Supplementary material

Supplementary Figure 1 **(A)** Histograms show HLA-A,B,C expression on indicated neuroblastoma cells lines before (blue; -IFNγ) or 24 h after treatment (red) with 0.1 ng/mL IFNγ (+IFNγ). An unstained sample (grey) is shown for comparison. **(B)** Representative flow cytometry histogram showing HLA-A*02 surface expression in parental LAN-1 cells before and after 24 h of IFNγ treatment. Each cell line shown in **(A)** and **(B)** was analyzed in one to three independent experiments. **(C)** Schematic representation of the second-generation L1CAM-CAR construct delivered using a third-generation lentiviral vector. Created in BioRender. Kuenkele, A. (2026) https://BioRender.com/jw4rew2.

Supplementary Figure 2 **(A)** NY-ESO-1 (gene name CTAG1B) expression in human neuroblastoma samples stratified by risk group, based on RNA-seq dataset GSE62564, assessed via the R2: Genomics Analysis and Visualization Platform (https://r2.amc.nl). Expression values represent log2-transformed normalized expression as provided by the dataset. **(B)** RNA expression of CTAG1B in LAN-1 neuroblastoma cells based on RNA-seq dataset GSE195577, accessed via the R2: Genomics Analysis and Visualization Platform. Expression values are shown as log2-normalized values as provided by the dataset. For comparison, CD8A and L1CAM are included as reference genes representing absent and highly expressed transcripts in this cell line, respectively. **(C)** qRT-PCR analysis of NY-ESO-1 (CTAG1B) expression in the parental LAN-1 cell line. 28S rRNA was amplified as a housekeeping/reference control to assess RNA input and cDNA quality, (n = 2 independent biological replicates, each derived from an independently cultured sample and analyzed in technical triplicates). **(D)** Schematic workflow for the production of NY-ESO-1 TCR T cells. Human CAR T cells for *in vivo* experiments were generated similarly, except that CD3+ instead of CD8+ T cells were used and expanded in the presence of 0.5 ng/mL IL-15 and 10 ng/µL IL-7. **(E)** Representative example of NY-ESO-1 TCR expression before and after MACS-mediated enrichment, assessed by flow cytometry using an antibody against the murine TCR β constant region (Trbc). **(F)** Summary of NY-ESO-1 TCR expression before and after MACS-mediated enrichment. Data are from four independent experiments using different T cell donors.

Supplementary Figure 3 **(A)** L1CAM-CAR knock-in efficiency was assessed by flow cytometry using an antibody targeting the CAR scFv. Data represent one representative example out of three independent experiment using T cells from different T cell donors. **(B)** CD4/CD8 ratio of L1CAM-CAR knock-in T cells at the end of the manufacturing process. Data are obtained from three independent experiments. Each symbol represents an individual T cell donor. Statistical significance was assessed using paired, two-tailed t-test. **(C)** Representative histograms showing L1CAM expression in parental LAN-1 and LAN-1 L1CAM KO cells. **(D)** Histogram showing HLA-A*02 expression on parental LAN-1 and LAN-1 L1CAM KO cells with or without IFNγ treatment. Numbers indicate mean fluorescent intensity (MFI) values, (n = 1). **(E)** Growth kinetics determined by Incucyte-based measurements of parental LAN-1 and LAN-1 L1CAM KO cells (the mean of 4 to 6 technical replicates is shown per biological replicate). Cell expansion is shown as log_2_-transformed fold change relative to baseline to facilitate comparison of growth kinetics between condition. Statistical significance was determined by two-way ANOVA with repeated measures and Greenhouse-Geisser correction, followed by Šídák’s multiple-comparison test. **(F)** Viability of LAN-1 cells after exposure to IFNγ. **(G)** Growth kinetic of LAN-1 L1CAM KO cells (n = 1), and parental LAN-1 cells (n = 1), with or without IFNγ treatment (10 ng/mL). Data are presented as mean ± SEM of four technical replicates from one biological replicate. **(H)** Histogram showing L1CAM expression on parental LAN-1 and LAN-1 L1CAM KO cells with or without IFNγ treatment. Numbers indicate mean fluorescent intensity (MFI) values, (n = 1). **(I)** qRT-PCR analysis of NY-ESO-1 (CTAG1B) expression in parental LAN-1 and LAN-1 L1CAM-KO cells. Expression was normalized to 28S rRNA and calculated by the ΔΔCt method using parental LAN-1 as calibrator. **(J)** Gating strategy used for the assessment of CD137 and CD25 expression in NY-ESO-1 TCR T cells shown in **Figure 3D**.

Supplementary Figure 4 **(A)** Parental LAN-1 and LAN-1 L1CAM KO cells were transduced with nuclear localization signal (NLS)-mKate or NLS-GFP, and expression of mKate and GFP was assessed by flow cytometry. **(B)** Gating strategy used to differentiate among the different cell population in the coculture experiment shown in **Figure 4**. NY-ESO-1 TCR T cells: CD3+, CTV-, Trbc+, mKate -, GFP -; L1CAM-CAR T cells: CD3+, CTV+, mKate-, GFP-; parental LAN-1: CD3-, CTV-, mKate+, GFP-; LAN-1 L1CAM KO: CD3-, CTV-, mKate-, GFP +.

Supplementary Figure 5 Exhaustion and phenotypic marker expression on L1CAM-CAR and untransduced T cells following expansion using the rapid expansion protocol (REP): **(A)** CD3^+^ T cells were either engineered to express an L1CAM-specific CAR or left untransduced. Following initial expansion and enrichment for CAR-expressing cells, T cells underwent a second expansion using the REP. Exhaustion marker expression in L1CAM-CAR or untransduced CD3+ T cells was assessed at the end of the REP, either directly or after three rounds of stimulation with L1CAM^+^ tumor cells. Data are from one experiment using T cells from the same donor. **(B)** Phenotypic marker expression on T cells with or without undergoing REP. All cells expressed the FAS receptor. The schematic on the right depicts the T cell subsets defined by the expression patterns of the indicated phenotypic markers. Note: T cells without (w/o) and after the REP were derived from different donors and were analyzed in individual experiments (n = 1). **(C)** Schematic representation of the murinized second-generation GD2-CAR construct. The congenic marker Thy1.1 was included as a non-immunogenic marker for identification of transduced cells. Created in BioRender. Kuenkele, A. (2026) https://BioRender.com/jw4rew2.
